# Supplementary material for: Unveiling the relationships between eco-anxiety, psychological symptoms and anthropocentric narcissism: The psychometric properties of the Turkish version of the Hogg eco-anxiety scale
Source: Glob Ment Health (Camb). 2024 Feb 14;11:e26. doi: 10.1017/gmh.2024.20 (PMC10988136; doi:10.1017/gmh.2024.20)
Supplement: Çimşir et al. supplementary material [file S2054425124000207sup001.docx]

**Supplementary Materials**

*Table S1. Original English Items of the Hogg Eco-Anxiety Scale (HEAS-13) and their Turkish Translation.*

| **Original English HEAS-13 Turkish translation of the HEAS-13** | |
| --- | --- |
| Over the last 2 weeks, how often have you been bothered by the following problems, when thinking about climate change and other global environmental conditions (e.g., global warming, ecological degradation, resource depletion, species extinction, ozone hole, pollution of the oceans, deforestation)?  1. Feeling nervous, anxious, or on edge  2. Not being able to stop or control worrying  3. Worrying too much  4. Feeling afraid  5. Unable to stop thinking about future climate change and other global environmental problems  6. Unable to stop thinking about past events related to climate change  7. Unable to stop thinking about losses to the environment  8. Difficulty sleeping  9. Difficulty enjoying social situations with family and friends  10. Difficulty working and/or studying  11. Feeling anxious about the impact of your personal behaviors on the earth  12. Feeling anxious about your personal responsibility to help address environmental problems  13. Feeling anxious that your personal behaviors will do little to help fix the problem  Response scale: 0 = not at all, 1 = several of the days, 2 = over half the days, 3 = nearly every day. | İklim değişikliği veya diğer çevresel koşullara bağlı olarak küresel ısınma, ekolojik dengenin bozulması, doğal kaynakların tükenmesi, türlerin yok olması, ozon tabakasının delinmesi, okyanusların kirlenmesi veya ormanların yok olması gibi durumlar konusunda aşağıdaki sorunları son iki haftalık süreyi dikkate alarak ne sıklıkla yaşadığınızı belirtiniz.  1.Gergin, kaygılı veya diken üstünde hissetmek  2.Kaygılanmayı durduramamak veya kontrol edememek  3.Aşırı kaygılanmak  4.Korku hissetmek  5.Gelecekteki olası iklim değişikliği veya diğer çevresel sorunlar hakkında düşünmeden edememek  6.İklim değişikliği ile ilgili geçmişte olanları düşünmeden edememek  7.Doğal çevreyle ilgili kayıpları düşünmeden edememek  8.Uyumakta zorlanmak  9.Aile ve arkadaşlarla yapılan sosyal etkinliklerinden keyif almakta zorlanmak  10.İşte ve/veya ders çalışırken zorlanmak  11.Kendi davranışlarınızın çevre üzerindeki etkileri konusunda endişe duymak  12.Çevre sorunlarını çözmeye yardımcı olmak için aldığınız kişisel sorumluluklarınız konusunda endişe duymak  13.Çevresel problemlerin çözülmesinde kişisel çabalarınızın yetersiz kalacağı yönünde endişe duymak  Yanıt ölçeği: 0 = hiç, 1 = bazı günler, 2 = pek çok gün, 3 = neredeyse her gün. |

HEAS-13 comprises four subscales: affective symptoms (true mean of items 1–4), rumination (true mean of items 5–7), behavioral symptoms (true mean of items 8–10), anxiety about personal impact (true mean of items 11–13)

*Table S2. Item Factor Loadings* *for the Anthropocentric Narcissism Scale (ANS)*

| **ITEMS** | **EFA** |
| --- | --- |
| 1. Compared to other living beings, we, humans, are the most valuable creatures in nature. | 0.661 |
| 2. Compared to other living beings, I think we humans have more rights over nature. | 0.686 |
| 5. I believe all other things (both living and non-living) in nature exist for our (humans’) well-being. | 0.626 |
| 6. As humans, I believe that we should dominate nature and other living things rather than adapt to them. | 0.649 |
| 7. I see us, humans, as the natural rulers/leaders of nature and other living beings. | 0.640 |
| 10. The importance of other living things and natural resources can be measured by the benefits they provide to humans. | 0.560 |
| 12. I believe that the world is a place that belongs to us, humans, more than any other living being. | 0.615 |
| Percentage of variance | 48.72 |
| Eigenvalue | 3.40 |
| Cronbach’s alpha | 0.82 |
| Composite reliability | 0.84 |

*Note.* EFA=exploratory factor analysis;

**Supplementary Note: Exploratory Factor Analysis of the ANS (Sample one)**

Because the construct of anthropocentric narcissism has qualities that are similar to narcissistic personality traits and human-centered environmental attitudes and actions, the initial pool of items was based on the theoretical and operational definitions associated with these constructs (e.g., Crowe et al., 2019; Dunlap et al., 2000). The initial list included 15 items, the face validity and theoretical relevancy of which were assessed by two scholars, with an interest in both personality and environmental issues. This process resulted in the retainment of 12 items, which were piloted on two individuals, a college student, and a colleague. Based on the feedback, minor changes were made to two of the items to guarantee item clarity. The resulting 12-item scale was entered into an EFA, which was conducted with the aim of revealing the factor structure of the ANS.

The factor structure of the ANS items was assessed using PAF, which is suggested as more robust for EFA (Costello & Osborne, 2005). The initial PAF indicated the reliability of the potential factors, yielding a Bartlett’s test (Chi-square = 1135,142, df = 66, p < 0.001) and a Keiser-Meyer-Olkin (KMO = 0.87). Three factors with eigenvalues greater than one were indicated by the analysis, with eigenvalues of 4.25 (35.40%), 1.19 (9.99%), and 1.08 (9.02%). A parallel analysis was run to make a sound decision regarding the number of factors (Costello & Osborne, 2005), which showed that only one factor had an eigenvalue that exceeds a chance value (O’Connor, 2000). This led us to decide on the single-factor solution, which was also compatible with our theoretical perspective of the construct of anthropocentric narcissism. As a result, the decision of the single-factor solution was based on the eigenvalue-greater-than-one rule, the result of the parallel analysis, and our theoretical perspective of the construct of anthropocentric narcissism

Following the retention of the single-factor solution, another PAF was conducted (Costello & Osborne, 2005). Item deletion was performed with the following criteria: (a) loading of an item on a factor is ≥ 0.40 (Costello & Osborne, 2005); (b) items have item-to-total correlations of more than 0.30 (Tabachnic & Fidell, 2007); and (c) communality values are more than 0.30 (Zeller, 2005). Items 4 and 11 were initially eliminated for not having a minimum communality value of 0.30 and a factor loading of 0.40. Items 8, 3, and 9 were consecutively eliminated for not having a minimum communality value of 0.30, decreasing the total number of remaining items to 7. The final PAF, with the remaining 7 items loading onto one factor, accounted for 48.72% of the variance in the construct of anthropocentric narcissism with an eigenvalue of 3.40. The resulting scale had a Cronbach’s alpha coefficient of 0.82 and a composite reliability score of 0.82. The items and the loadings of the final version are shown in Table S2.

*Table S3. Item Level Descriptive Statistics of the Turkish Version of the HEAS-13*

| Item | Mean | Std. Dev. | Skewness | Kurtosis |
| --- | --- | --- | --- | --- |
| 1 | 1.2 | 0.70 | 0.59 | 0.47 |
| 2 | 0.77 | 0.72 | 0.68 | 0.19 |
| 3 | 0.76 | 0.75 | 0.84 | 0.49 |
| 4 | 0.89 | 0.71 | 0.56 | 0.37 |
| 5 | 1.1 | 0.79 | 0.35 | -0.24 |
| 6 | 0.90 | 0.76 | 0.49 | -0.21 |
| 7 | 1.2 | 0.77 | 0.32 | -0.17 |
| 8 | 0.72 | 0.88 | 1.1 | 0.25 |
| 9 | 0.70 | 0.72 | 0.79 | 0.16 |
| 10 | 0.91 | 0.89 | 0.70 | -0.31 |
| 11 | 1.13 | 0.84 | 0.45 | -0.30 |
| 12 | 0.99 | 0.73 | 0.31 | -0.28 |
| 13 | 1.34 | 0.87 | 0.14 | -0.65 |

*Table S4. Means and Standard Deviations for the HEAS-13 Subscales in New Zealand, Australia, Italy and Turkey*

|  | **New Zealand**  **(**[**Hogg et al., 2021**](https://www.sciencedirect.com/science/article/pii/S0272494423002281#bib27)**)** | **Australia (Hogg et al., 2023)** | **Italy**  **(Rocchi et al., 2023)** | **Turkey** |
| --- | --- | --- | --- | --- |
|  | M(SD) | M(SD) | M(SD) | M(SD) |
| Affective Symptoms | 0.66(0.79) | 0.88(0.80) | 0.98(0.77) | 0.92(0.59) |
| Rumination | 0.33(0.59) | 1.08(0.90) | 0.88(0.81) | 1.10(0.69) |
| Behavioral Symptoms | 0.63(0.80) | 0.58(0.72) | 0.45(0.63) | 0.79(0.76) |
| Anxiety about Personal Impact | 0.91(0.64) | 1.39 (0.93) | 1.42(0.87) | 1.16(0.66) |
